# Supplementary material for: MetaQTL: a package of new computational methods for the meta-analysis of QTL mapping experiments
Source: BMC Bioinformatics. 2007 Feb 8;8:49. doi: 10.1186/1471-2105-8-49 (PMC1808479; doi:10.1186/1471-2105-8-49)
Supplement: Additional File 7 — MetaQTL Package : jar file and tutorial. This Zip archive contains both the MetaQTL JAR file and the files of the tutorial. [file 1471-2105-8-49-S7.zip › org.inra.metaqtl/doc/org/metaqtl/Qtl.QTLProj.html]

Qtl.QTLProj


|  |  |  |
| --- | --- | --- |
| **Overview** \| **Package** \| **Class** \| **Use** \| **Tree** \| **Deprecated** \| **Index** \| **Help** | |  |
| **PREV CLASS**   **NEXT CLASS** | **FRAMES**    **NO FRAMES**     **All Classes** |  |
| SUMMARY: NESTED \| FIELD \| CONSTR \| METHOD | DETAIL: FIELD \| CONSTR \| METHOD |  |


---


## org.metaqtl Class Qtl.QTLProj

```
java.lang.Object
  org.metaqtl.Qtl.QTLProj
```

**All Implemented Interfaces:**: java.io.Serializable

**Enclosing class:**: Qtl

---

``` public class Qtl.QTLProj extends java.lang.Object implements java.io.Serializable ```

This class stores the QTL projection
properties.

**See Also:**: Serialized Form

---

| **Field Summary** | |
| --- | --- |
| `double` | `intScale`             The scale ratio between the original flanking marker interval and the one on the map used for projection. |
| `java.lang.String` | `mapOrigin`             The name of the map from which this QTL comes. |
| `double` | `mapScale`             The scale ratio between the original map and the map on which the QTL has been projected. |
| `double` | `mapWeight`             The cumulated scale weights of the common marke sequences between the original map and the map on which the QTL has been projected. |
| `int` | `o_mrkIdxL`             The original indice of the left flanking marker. |
| `int` | `o_mrkIdxR`             The original indice of the right flanking marker. |
| `int` | `s_mrkIdxL`             The skeleton indice of the left flanking marker. |
| `int` | `s_mrkIdxR`             The skeleton indice of the right flanking marker. |
| `boolean` | `shareFlanking`             True if the two maps share a common marker sequence around the QTL. |
| `boolean` | `swap`             True if the flanking marker order are inversed between the skeleton and the original map. |


**Constructor Summary**

`Qtl.QTLProj()`


| **Method Summary** | |
| --- | --- |
| `Qtl.QTLProj` | `getCopy()` |

**Methods inherited from class java.lang.Object**

`clone, equals, finalize, getClass, hashCode, notify, notifyAll, toString, wait, wait, wait`

**Field Detail**

### mapOrigin

```
public java.lang.String mapOrigin
```

:   The name of the map from which
    this QTL comes.

---


### o\_mrkIdxL

```
public int o_mrkIdxL
```

:   The original indice of the left
    flanking marker.

---


### o\_mrkIdxR

```
public int o_mrkIdxR
```

:   The original indice of the right
    flanking marker.

---


### s\_mrkIdxL

```
public int s_mrkIdxL
```

:   The skeleton indice of the left
    flanking marker.

---


### s\_mrkIdxR

```
public int s_mrkIdxR
```

:   The skeleton indice of the right
    flanking marker.

---


### mapScale

```
public double mapScale
```

:   The scale ratio between the original
    map and the map on which the QTL has
    been projected.

---


### mapWeight

```
public double mapWeight
```

:   The cumulated scale weights of the common
    marke sequences between the original map
    and the map on which the QTL has been projected.

---


### intScale

```
public double intScale
```

:   The scale ratio between the original flanking
    marker interval and the one on the map used
    for projection.

---


### shareFlanking

```
public boolean shareFlanking
```

:   True if the two maps share a common marker sequence
    around the QTL.

---


### swap

```
public boolean swap
```

:   True if the flanking marker order are inversed between
    the skeleton and the original map.


**Constructor Detail**

### Qtl.QTLProj

```
public Qtl.QTLProj()
```


**Method Detail**

### getCopy

```
public Qtl.QTLProj getCopy()
```

:   **Returns:**


---


|  |  |  |
| --- | --- | --- |
| **Overview** \| **Package** \| **Class** \| **Use** \| **Tree** \| **Deprecated** \| **Index** \| **Help** | |  |
| **PREV CLASS**   **NEXT CLASS** | **FRAMES**    **NO FRAMES**     **All Classes** |  |
| SUMMARY: NESTED \| FIELD \| CONSTR \| METHOD | DETAIL: FIELD \| CONSTR \| METHOD |  |


---
